# Distinct Abnormalities of Small Bowel and Regional Colonic Volumes in Subtypes of Irritable Bowel Syndrome Revealed by MRI

**DOI:** 10.1038/ajg.2016.538

**Published:** 2016-12-13

**Authors:** Ching Lam, Gemma Chaddock, Luca Marciani Laurea, Carolyn Costigan, Eleanor Cox, Caroline Hoad, Susan Pritchard, Penny Gowland, Robin Spiller

**Affiliations:** 1 NIHR Biomedical Research Unit in Gastrointestinal and Liver Diseases, Nottingham University Hospitals NHS Trust and the University of Nottingham, Nottingham, UK; 2 Sir Peter Mansfield Imaging Centre, University of Nottingham, Nottingham, UK

## Abstract

**OBJECTIVES:**

Non-invasive biomarkers which identify different mechanisms of disease in subgroups of irritable bowel syndrome (IBS) could be valuable. Our aim was to seek useful magnetic resonance imaging (MRI) parameters that could distinguish each IBS subtypes.

**METHODS:**

34 healthy volunteers (HV), 30 IBS with diarrhea (IBS-D), 16 IBS with constipation (IBS-C), and 11 IBS with mixed bowel habit (IBS-M) underwent whole-gut transit and small and large bowel volumes assessment with MRI scans from *t*=0 to *t*=360 min. Since the bowel frequency for IBS-M were similar to IBS-D, IBS-M and IBS-D were grouped together and labeled as IBS non-constipation group (IBS-nonC).

**RESULTS:**

Median (interquartile range): fasting small bowel water content in IBS-nonC was 21 (10–42), significantly less than HV at 44 ml (15–70), *P*<0.01 as was the postprandial area under the curve (AUC) *P*<0.01. The fasting transverse colon volumes in IBS-C were significantly larger at 253 (200–329) compared with HV, IBS-nonC whose values were 165 (117–255) and 198 (106–270) ml, respectively, *P*=0.02. Whole-gut transit time for IBS-C was prolonged at 69 (51–111), compared with HV at 34 (4–63) and IBS-D at 34 (17–78) h, *P*=0.03. Bloating score (VAS 0–10 cm) correlated with transverse colon volume at *t*=405 min, Spearman *r*=0.21, *P*=0.04.

**CONCLUSIONS:**

The constricted small bowel in IBS-nonC and the dilated transverse colon in IBS-C point to significant differences in underlying mechanisms of disease.

## INTRODUCTION

Irritable bowel syndrome (IBS) is ubiquitous and yet our knowledge of the mechanisms underlying this condition remained limited. Treatment for IBS is unsatisfactory as it is based on patient history, which can be unreliable. Thus recording stool patterns by daily diary show most patients overestimate how abnormal their bowel patterns are (1). Objective markers could be valuable if they allowed a categorization based on abnormalities of pathophysiology. The ideal would be non-invasive and patient acceptable. This unfortunately is not true of manometry and barostat studies while gamma scintigraphy and abdominal x-rays, although non-invasive, involve exposure to radiation, which would not be ideal in this cohort of patients as they comprise mostly young women. Therefore, there is a need for objective measures of small bowel and colonic function in this group of patients to guide treatment.

Magnetic resonance imaging (MRI) has many advantages because of its ability to study the gastrointestinal physiology and its function without exposure to ionizing radiation. It has been used to noninvasively measure small (2) and large bowel (3) volumes both fasting and fed in health and IBS and also to directly visualize and measure colonic wall movements (4). Previous studies showed distinctive abnormalities in IBS patients with diarrhea (IBS-D) with decreased small bowel water content (SBWC) (2) and an ascending colon (AC), which did not show the usual postprandial increase but instead showed an immediate increase in transverse colon (TC) volume suggesting failure of postprandial accommodation in the AC (3). It is unclear whether this is a feature of all IBS or specifically to IBS-D. Therefore, our aim in this study was to assess small bowel, colonic regional volumes and gut transit in subtypes of IBS to determine if there are distinct regional differences in the different small intestine and colon of the three main subtypes of IBS.

## METHODS

This was an open label study designed to compare the underlying pathophysiology of the small and large bowel in different subtypes of IBS. The study was approved by the National Research Ethics Committee, United Kingdom (11/EM0245). This study was registered with the Clinicaltrials.gov (Identifier NCT01534507). All subjects gave written informed consent and the study was carried out according to the Good Clinical Practice principles.

### Subjects

Ninety-one subjects took part in the study. Thirty-four subjects were healthy volunteers (HV; 15 female, 19 male, 18–70 years) free from any history of gastrointestinal disease and surgery. Data for 21 of the 34 HV were obtained from a previous study (5), which had similar MRI protocol and study design as current study but only the transit times have been published and these are now reported here. Fifty-seven subjects diagnosed by a doctor as having IBS were recruited from general gastroenterology clinics or via social media advertisements. The IBS group comprised of the following subtypes: 30 IBS with diarrhea (IBS-D) (18 female, male, 19–72 years), 16 IBS with constipation (IBS-C; 15 female, 1 male, 18–56 years) and 11 IBS with mixed bowel habit (IBS-M; 7 female, 4 male, 20–62 years). All these 57 subjects satisfied the Rome III criteria for IBS. A paper stool diary completed 7 days before the study was used to subtype their IBS based on stool form and frequency (6) (IBS-C=>25% type 1 or 2 and <25% type 6 or 7, IBS-M=>25% type 1 or 2 and >25% type 6 or 7, IBS-D=>25% type 6 or 7 and <25% type 1 or 2). Patients classified as un-subtyped by stool diary were not included in the study. All subjects were required to stop any medications likely to affect bowel habit 14 days before the study, as well as avoid certain food known to aggravate IBS symptoms. All subjects completed a Hospital Anxiety and Depression (HAD) (7), Patient Healthy Questionnaire-12 Somatic Symptoms (PHQ12SS) (8), Visceral sensitivity index (VSI) (9), IBS severity score (IBSSS) (10) and Perceived Stress Scale (11) questionnaires to assess psychological and severity of IBS symptoms. A MRI safety questionnaire was also completed to exclude contraindications to MRI.

### Study design

**Figure 1:**
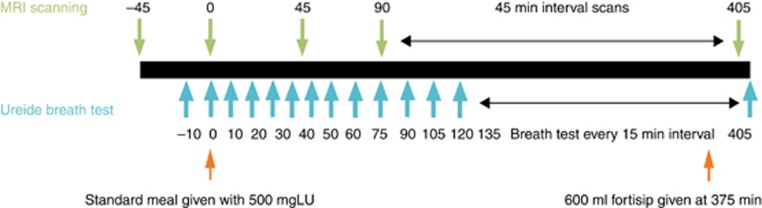
Summary of study day. A full color version of this figure is available at the *American College of Gastroenterology* journal online.

The subjects swallowed 5 MRI marker capsules between 08:00–09:00 hours the day before the study day, to measure whole-gut transit time (WGTT) as previously described (5). The subjects were reminded by telephone to swallow the 5 MRI marker capsules a day prior to the study day. Orocecal transit time (OCTT) was assessed using the Lactose-C13 Ureide breath test (LUBT) as previously described (5, 12). The subjects were asked to ingest 1 g (6 mmol) of unlabeled lactose ureide (Euriso-top, St Aubin Cedex, France) three times during the day preceding the scans along with meals (morning, afternoon, and evening), to stimulate bacterial enzyme activity to cleave the lactose ureide in the colon. The following day, the subjects had a baseline MRI scan and provided a breath sample before consuming a 362 kcal breakfast which consisted of 220 g creamed rice pudding (J Sainsbury plc, London, UK) mixed with 34 g strawberry jam (J Sainsbury plc), 15 g course wheat bran (Holland and Barrett, Hinkley, UK) and 500 mg C^13^ labeled Lactose Ureide along with100 ml orange juice (J Sainsbury plc). Following this, the subjects were scanned every 45 min for 7.5 h. Subjected were required to provide a breath sample every 10 min for the first hour and then every 15 min for the next 7 h to assess OCTT. Approximately 6 h after breakfast, the subjects were fed a mixed nutrient meal consisting of 600 ml of Fortisip drink (Nutricia, Trowbridge, UK; Figure 1). All subjects completed questionnaires scoring abdominal symptoms (fullness, bloating, distension, abdominal pain and nausea) on a 0–10 cm visual analogue scale (VAS) scale immediately following each MRI scan to correlate these symptoms with the MRI findings.

#### MRI protocol

MRI was carried out using a 1.5T Philips Achieva scanner (Philips Medical System, Best, The Netherlands). Subjects were scanned in the supine position using a 16-channel XL torso coil. Various imaging sequences were acquired to measure the following parameters: SBWC, AC water content, colonic volumes, colonic gas, and WGTT. A single-shot turbo-spin echo sequence (TR/TE=8000/320 ms, FA=90^0^, field of view=400 × 362 × 168 mm^3^, acquired resolution=1.56 × 2.9 × 7.0 mm^3^) was used to acquire T2 weighted coronal images of the abdomen for measurement of SBWC, as previously validated (13). A dual echo fast field echo sequence (TR/TE_1_/TE_2_=157/2.3/4.6 ms, FA=80^0^, FOV=450 × 362 × 168 mm^3^, acquired resolution=2.01 × 2.87 × 7 mm^3^), acquiring 24 coronal slices, was used to measure colonic volumes (3) and colonic gas (14). For assessment of WGTT, coronal scans were obtained at two stations with a 30 mm overlap using a multi-echo FFE sequence (TE_1_=1.41 ms, TE_2_=2.5 ms, TR=3.8 ms, FA=10^0^, FOV=250 × 371 × 200 mm^3^, AQR 1.8 × 1.8 × 3.6 mm^3^, SENSE factor=2). This sequence was used to create a maximum intensity projection image from water only reconstructed images, which allowed for three-dimensional visualization of the marker capsules as previously reported (5).

Each image set was acquired on an expiration breath-hold for up to 25 s, depending on the sequence. Subjects spent ~15 min inside the magnet per time point, and sat in an upright position in the waiting room between each scan.

### Data analysis

Measurements of colonic volumes were carried out by manually tracing around regions of interest (ascending, transverse, and descending colon) using Analyze9 software (Biomedical Imaging Resource, Mayo Foundation, Rochester, MN, USA). The software created three-dimensional object maps and summed volumes across all slices (3). Colonic gas was determined using these Analyze9 object maps using in-house software written in IDL (IDL 6.4, Research Systems, Boulder, CO, USA) (15). Colonic gas was qualitatively identified as regions that were completely black on the sum of the dual echo images. The distribution of noise in both echoes from the dual echo coronal images was determined by drawing regions of interest in the gas regions visible on the images. If no gas regions were visible the noise was determined from gas in the stomach or from noise regions outside the body. The colonic gas volume was then calculated as the voxel size multiplied by the number of voxels which were within the noise on both dual echo images (assessed for each echo separately) and which were located within the regions defined by the colonic volumes. An in-house program was used to measure SBWC using a previously validated method (13). Measurement of WGTT was determined by scoring the position of the 5 marker capsules 24 h after ingestion and using a previously validated algorithm to determine a WGT time in hours (5). Breath test samples were analyzed using an IRIS-Lab analyser machine (Wagner Analysen Technik, Bremen, Germany). The OCTT was taken as the time at which there is a rise in breath C^13^ which is 2.5 times the s.d. of all previous points, as defined in a previous validation study (5, 12).

### Power and statistical analysis

The primary end point of this study was to compare the fasting SBWC in three subtypes of IBS, compared with healthy subjects. Secondary endpoints include OCTT in three subgroups of IBS, area under the curve (AUC) post-prandial SBWC, colonic volumes both fasting and postprandial, WGTT and correlation between HAD and OCTT and WGTT.

Statistical analysis was carried out with the use of GraphPad Prism version 6.0 for Windows (GraphPad Software Inc, San Diego, CA, USA). Normality of the data was tested by using the D'Agostino and Pearson omnibus normality test. Normally distributed data are expressed as mean±s.d. and non-normally distributed data are expressed as median (interquartile range; IQR). Normally distributed data were analyzed using the unpaired *t*-test, one-way analysis of variance and two-way analysis of variance. *Post hoc* assessments were performed by using Bonferroni's multiple comparison test. Non-normally distributed data were analyzed using Mann–Whitney test and Kruskal–Wallis test. *Post hoc* assessments were performed by using Dunn's multiple comparison test. In the *post hoc* assessments, result were considered significant if *P*≤0.03, thus accounting for the effect of multiple comparisons.

The sample size for the IBS-M group was very small (*n*=11). Their baseline demographics were listed in Table 1. As the bowel frequency for IBS-M were similar to IBS-D, we have grouped IBS-M and IBS-D together and labeled as ‘non-constipated IBS' (IBS-nonC) in subsequent analysis.

#### Sample size

Our previous study indicates the fasting SBWC in IBS-D was 50.6 (33.3) ml (mean (s.d.)), *n*=26 compared with normal values of 90.9 (67.7) ml, *n*=18 (pooled variance 1740) (2). Using *n*=30 in each group will give us a 90% power (*α*=0.05) to detect a mean difference 20.3 ml from HV, a difference which is around half that previously observed in IBS-D. Although we achieved our target for IBS-D we under-recruited for the other subtypes and so combined these two groups for analysis of the MRI parameters.

We had no MRI data on WGT when the study was set-up but previous work from the Mayo Clinic using geometric centre of isotope markers suggests that *n*=14 would be sufficient to detect clinically relevant effect sizes in the position of a group of markers in the colon at 24 or 48 h respectively (16). IBS-D predominated in the patients recruited from our clinics so in the event we over-recruited this subgroup but found IBS-M surprisingly infrequent and so under-recruited this subgroup.

## RESULTS

### Demographics

The IBS patients in this study as expected were predominately female and were significantly more anxious with high IBSSS and VSI scores compared with HV. They also had significantly more non-gastrointestinal, somatic symptoms with high PHQSS12 scores of 6 (4–9) when compared with HV of 2 (0–3), *P*<0.01 (Table 1). IBS patients had significantly more abdominal symptoms such as abdominal pain, urgency and bloating than HV (Table 1). As expected subdividing patients according to their stool diaries created groups with the predicted differences in bowel frequency and stool consistency. Both IBS-D and IBS-M had greater stool frequency and Bristol Stool form score than IBS-C. IBS-D had significantly more urgency than IBS-C.

### MRI results

#### Small bowel water content

**Figure 2:**
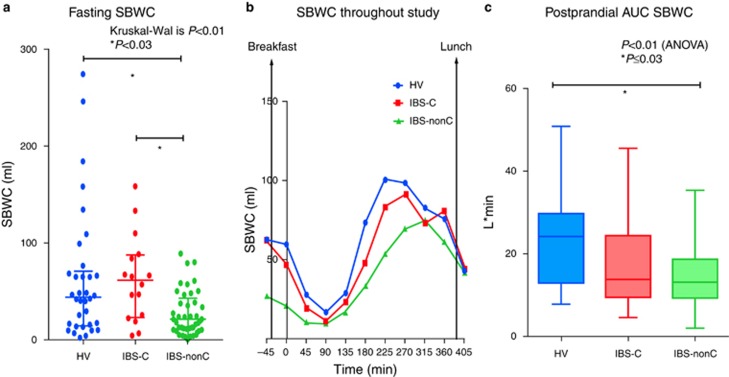
Showing significant differences in small bowel water (SBWC) between irritable bowel syndrome (IBS) subtypes and healthy volunteers (HV). (**a**) Fasting small bowel water showing IBS-nonC had significantly lower value. (**b**) SBWC throughout the whole study for IBS subtypes and HV confirming that the lower values in IBS-nonC persist throughout the postprandial period. (**c**) Area under the curve (AUC) for post-prandial small bowel water content (times between time *t*=0 to *t*=360 min) showing IBS-nonC have signficantly lower SBWC. A full color version of this figure is available at the *American College of Gastroenterology* journal online.

Fasting SBWC in IBS-nonC was significantly less than HV and IBS-C (*P*=0.03 and *P*<0.01 respectively; Table 2 and Figure 2a). As in previous studies the SBWC after our standard mixed solid/fluid meal showed a biphasic response with an initial rapid fall from time 0–90 min followed by a sustained rise from 90 to 270 min (Figure 2b). The area AUC for post-prandial SBWC was assessed between the times after completing the test meal (*t*=0 min) to the last MRI scans before lunch being provided (*t*=360 min). The postprandial AUCs for SBWC were significantly lower for IBS-nonC when compared with HV (Table 2 and Figure 2c). IBS-C patients in contrast were no different from the HVs in this respect.

#### Colonic volumes and gas

**Figure 3:**
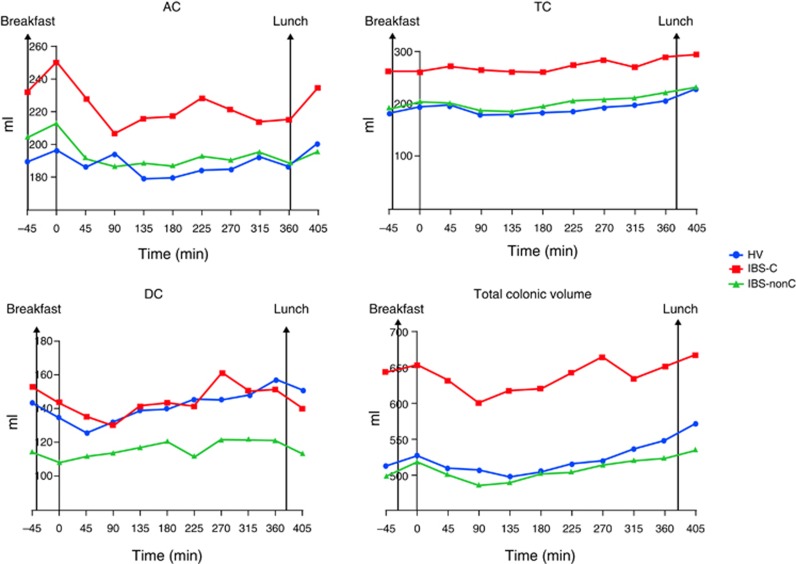
Segmented colonic volumes and total colonic volumes throughout whole study for irritable bowel syndrome (IBS) subtypes and healthy volunteers (HV) showing significantly greater total colonic volumes in IBS with constipation (IBS-C) mainly due to the significantly greater transverse colon (TC). A full color version of this figure is available at the *American College of Gastroenterology* journal online.

Fasting total colonic volumes in IBS-C were significantly larger than IBS-nonC and HV (Table 3 and Figure 3). Although fasting colonic segmental volumes (AC, TC and descending colon (DC)) were all numerically larger in IBS-C compared with HV only the TC volumes were significantly so. Immediately after eating (*t*=0 min), the AC volume showed a small rise in all but the IBS-D patients. This rise in AC volume was not significantly different between the 3 groups; *P*=0.15, Kruskal–Wallis. Following *t*=0 min, there were a decline in AC volume for all groups.

Postprandially, the TC volumes remained steady for the initial 3 h but then rose steadily over the subsequent 3 h. The TC colon was significantly greater than baseline at the end of study (*t*=405 min) for HV with mean difference 46 ml (standard error difference=22 ml), *P*=0.04. There were no significant changes in TC volumes at the end of study (*t*=405 min) compared with baseline fasting TC (*t*=−45 min) for the IBS-C (mean difference=3 ml (standard error difference=24 ml); *P*=0.9) and IBS-nonC (mean difference=9 ml (standard error difference=15 ml)).

**Figure 4:**
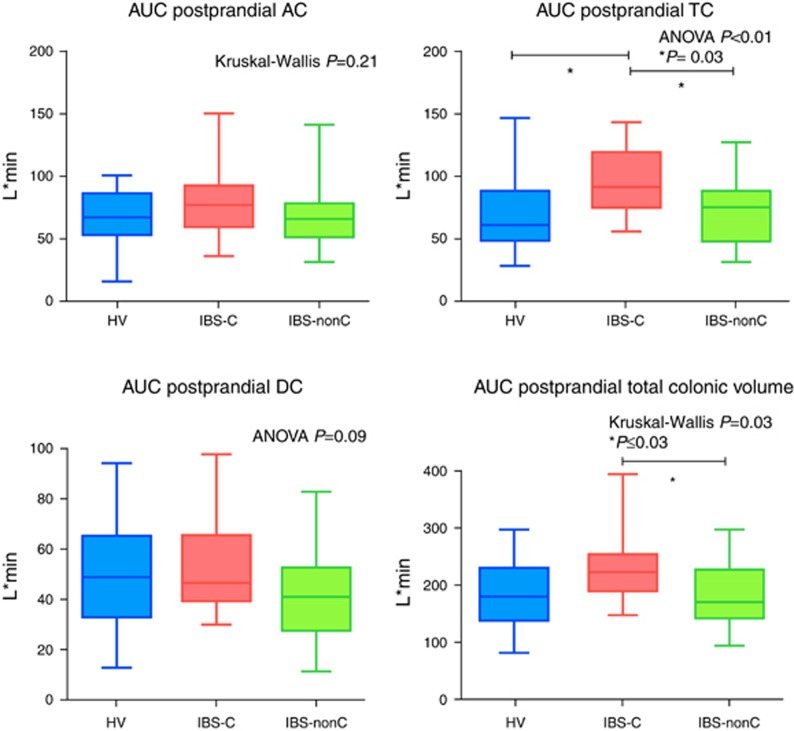
Area under the curve (AUC) of colonic volumes postprandially (times between *t*=0 to *t*=360 min) showing the significant increase in irritable bowel syndrome with constipation (IBS-C) largely due to the increased transverse colon (TC). A full color version of this figure is available at the *American College of Gastroenterology* journal online.

The AUC for TC in IBS-C was strikingly and significantly larger than HV and IBS-nonC and overall postprandial total colonic volume in IBS-C was also significantly larger compared with IBS-nonC (Table 3 and Figure 4). The fasting total colonic gas in all 3 groups was similar and overall AUC postprandial total colonic gas (*t*=0 to *t*=360 min) did not show significant difference in the three groups (Table 3).

#### Orocecal transit test using LUBT

The OCTT for HV and IBS subtypes are shown in Table 4. Even though IBS-D subtype had numerically lower OCTT, this was not statistically significant.

#### WGTT

**Figure 5:**
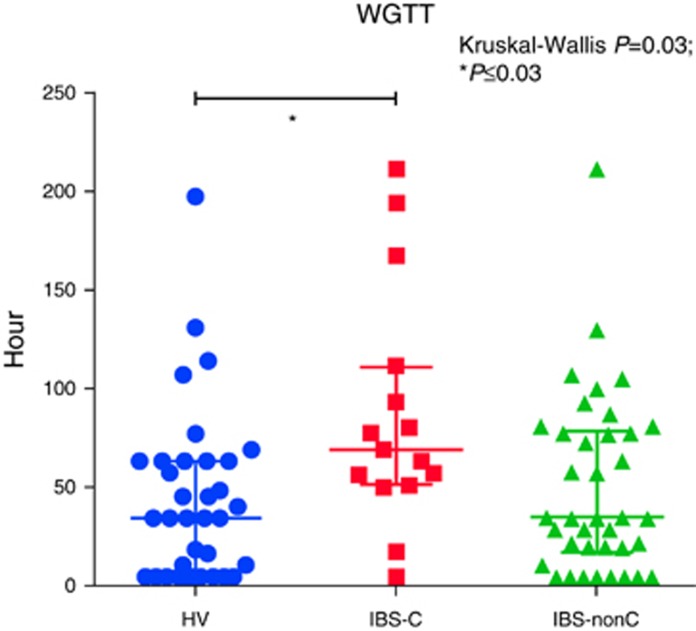
Whole-gut transit time between irritable bowel syndrome (IBS) subtypes and healthy volunteers (HV). A full color version of this figure is available at the *American College of Gastroenterology* journal online.

The WGTT using the MRI marker capsules showed the expected wide variability in IBS subtypes compared with HV (Table 4 and Figure 5). The WGTT for IBS-C was significantly longer than HV. The WGTT for IBS-nonC was not different from HV.

### Clinical symptoms

#### Correlations between anxiety, whole-gut transit and OCTTs

There was no correlation between anxiety and OCTT in IBS patients overall (Spearman *r*=− 0.18, *P*=0.1). Subgroup analysis showed this was only significant in the IBS-nonC patients (Table 5). There was a significant correlation between anxiety and WGTT in the IBS-C group whereas the other 2 IBS groups showed no correlation (Table 5).

#### Correlations between clinical symptoms of bloating, distension, fullness, pain, and nausea, and segmented colonic volumes at *t*=405 min

**Figure 6:**
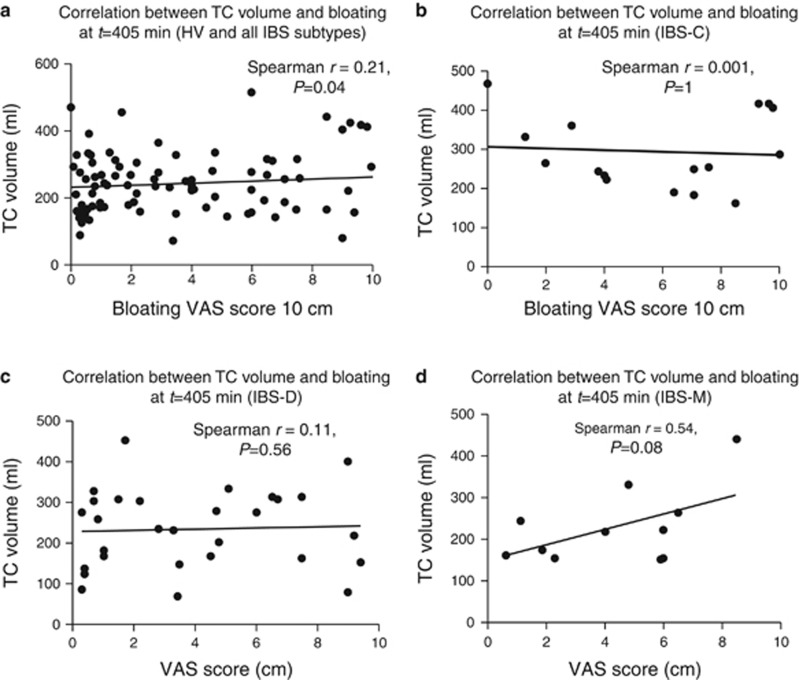
(**a**) Correlation between transverse colon (TC) volume and VAS bloating score at *t*=405 min for all subjects. (**b**) Correlation between TC volume and VAS bloating score at *t*=405 min for irritable bowel syndrome with constipation (IBS-C). (**c**) Correlation between TC volume and VAS bloating score at *t*=405 min for IBS with diarrhea (IBS-D). (**d**) Correlation between TC volume and VAS bloating score at *t*=405 min for IBS with mixed bowel habit (IBS-M).

There was weak but significant correlation between bloating score (VAS 0–10 cm) and TC volume following lunch at time *t*=405 min; Spearman *r*=0.21, *P*=0.04 (Figure 6). The bloating score was higher in IBS-C following lunch, at time *t*=405 min, being 6 (3.2–9.3) while for other IBS subtypes it was 3.7 (1.1–7.4) cm, however owing to wide variability this just failed to reach statistical significance *P*=0.06. There were weak correlations between distension, pain and nausea with TC volume at time *t*=405 min, however, none of these achieved statistical significance after correction for mulitple comparisons. There were no correlations between fasting SBWC with symptoms such as bloating, distension, fullness, pain, and nausea.

## DISCUSSION

Objective assessments of the undisturbed small bowel and colonic regional volumes have not been previously possible. Using MRI we have been able to provide much unique data emphasizing the differences in small and large bowel volumes and motility in IBS subtypes. Our major finding was the striking increase in TC volumes in IBS-C compared with both healthy controls and IBS-nonC, both fasting and postprandially (see Supplementary Figure S1 online). The TC may act as a buffer accommodating material from the AC and the descending colon whose volumes can change rapidly following a meal and defecation. Thus we have previously reported the normal initial rise in AC volume of 20–50 ml within 30 min after a meal as the gastro-ileal reflex empties residue from the last meal into the colon before the arrival of the next meal. This rise is followed by a fall in the first couple of hours after a meal. The opposite trend is seen with the descending colon whose volume falls immediately after a meal in healthy as well as IBS-nonC (3). This fall may reflect retrograde movement from the sigmoid and left colon into the TC as has previously been reported postprandially (17). Interestingly the postprandial changes in the descending colon did not seem as obvious with IBS-nonC compared with HV and IBS-C groups suggesting less retrograde movements which supports previous scintigraphic work (18). Thus the fall in both AC and descending colon volumes seen in IBS-C resulted in a more prolonged rise in TC volumes, which accommodates this extra load. The TC shape is highly variable and can extend down into the pelvis allowing it to increase its volume making it well designed to perform as a variable storage area.

Bloating is a very common sensation but poorly understood. Some individuals physically distend and although the sensation of bloating can come on at any time abdominal distension generally increases over the day (19). The sensation of bloating showed similar biphasic response to meals which increased after breakfast (*t*=0 min) and then slowly declined with a second peak immediately after eating the second meal at *t*=405 min. The bloating score and the TC volume following lunch (*t*=405 min) showed a significant correlation between each other. Our IBS-C patients had a higher bloating VAS score than other IBS subtypes as others have found (19). Bloating correlated better with distension of the TC than AC, suggesting that the TC is more sensitive than the AC in keeping with a general gradient of increasing sensitivity as one moves distally in the gut. We had predicted that it would be gas in the TC that caused a larger TC volume but this was not so. The total gas volume detectable with our technique in the TC at *t*=405 min amounted to only 13 (1–50) ml in IBS-C with similarly small amount of colonic gas in HV, IBS-D and IBS-M giving values of 17 (2–33), 10 (1–45) and 2 (0–5) ml respectively, *P*=0.11).

Unexpectedly the AUC of the postprandial total colonic gas in HV was larger than in the IBS subtypes yet these volumes in HV do not correlate with bloating symptom (Spearman *r*=−0.2, *P*=0.32). The median peak bloating VAS score was minimal at 1.4 cm for the HV during the study. This insensitivity to distension in HV supports our previous findings that even quite marked distension of the AC following ingestion of an osmotic laxative, produced virtually no abdominal symptoms in HV (20) presumably because there is receptive relaxation of the colonic muscle and hence no rise in wall tension which is what drives symptoms in both stomach (21) and rectum (21, 22). This may explain why smooth muscle relaxants improve IBS symptoms in some patients (23).

The fasting SBWC for IBS-nonC was significantly smaller than HV, a finding consistent with our previous study in a separate IBS-D cohort (2). The IBS-M group had similar results as the IBS-D group probably because they were in their ‘diarrheal' phase around the study day since their baseline average stool frequency during the study period was similar to the IBS-D group (Table 1). The smaller fasting SBWC values in IBS-nonC distinguish them from both healthy controls and IBS-C. Our previous study had observed narrowed small bowel in IBS-D patients which we called the ‘spaghetti bowel'. This correlated inversely with anxiety and this may reflect increased muscular tone in small bowel rather than accelerated transit (2). We were able to reproduce this feature in HVs using an injection of the stress hormone Corticotrophin Releasing Factor (24) suggesting this might be a stress response in susceptible subjects.

We used the LUBT method to measure OCTT which has been shown to correlate with scintigraphic assessment with a slight overestimate (12). We found no correlation between fasting SBWC and OCTT nor between OCTT and postprandial AUC SBWC.

The OCTT and WGTT for IBS subtypes show a range of values for all subgroups mostly within the wide normal ranges for OCTT. This is one of the few studies where LUBT was used to assess OCTT in IBS. Unlike the lactulose breath hydrogen method in which the 10 g of lactulose used may accelerate small bowel transit, the amount of lactose-ureide used (1 g) in this study was very small and unlikely to alter OCTT. Our OCTT values were slightly shorter than the study in HVs by Geypens *et al.* (12) which may be due to the different meal composition.

The WGTT in IBS-C as others have found (25, 26) was significantly greater than HV whereas the WGTT in IBS-nonC was similar to HV (*P*=0.60). The fast WGTT (median (IQR)=34 (19–81) h) in IBS-M is in keeping with their increased average bowel frequency compared to HV (Table 1).

### Limitations

Although we were adequately powered for the IBS-D group the numbers in IBS-C and IBS-M groups were lower than planned. The clinical and MRI features for IBS-M were similar, therefore they were grouped together with IBS-D. One problem with IBS-M is knowing when to study them. It seems likely that the MRI features we described would be different during different phases of their condition. Perhaps it is their variability, which is the key feature, which means they need to be studied on at least 2 occasions to capture this aspect. It should be recognized that the method used for WGTT has only been validated against the gold standard radio-opaque method for values in the range 0–80 h. Thus while we can be confident in the values for most of the patients in the present study we can be less certain about the few with WGTT over 80 h although we can be confident they are >80 h. We, using nonparametric statistics, which do not rely on a linear scale to assess the findings that IBS-C, have prolonged transit so we feel confident that these limitations have not invalidated our conclusions. The typical wide variability both inter-individual and intra-individual in both our and others studies likely reflect true variability induced by daily differences in diet, emotions and defecatory timings in both patients and HV which are not usually controlled as was true in our study.

We have not adjusted the *P* values for the multiple comparisons shown in Table 3. Although this risks increasing the Type 1 error rate the differences we have observed are not random but all fit the same pattern, namely that IBS-C have slower transit and larger colons so we believe a Bonferroni correction would be over conservative. However we do accept that our findings need confirmation in a separate cohort.

The typical wide variability both inter-individual and intra-individual in both our and others studies likely reflect true variability induced by daily differences in diet, emotions and defecatory timings in both patients and HV which are not usually controlled as was true in our study. Another limitation was the lack of recording of bowel symptoms during the study period. The bowel movements for each participant may vary with each IBS subtypes and thus affect its colonic volumes throughout the day however we found the postprandial AUC for the total colonic volumes between HV, IBS-D, and IBS-M were similar and did not show the abrupt fall in volume we would have anticipated had defaecation occurred.

This is the first study using MRI as a tool to give us a better insight into different gastrointestinal physiology of IBS patients. One of our aims was to determine whether colonic volumes and SBWC could be better biomarkers for IBS subtypes than symptoms alone. Using the fasting TC volume there was quite an overlap between the constipated and non-constipated IBS. However, using a cut-off for the TC volume of 300 ml, only 31% IBS-C meet this criteria but specificity would be 95%. This defines a distinct subgroup which have a significantly enlarged TC. Whether such individuals respond differently to treatments is something we aim to evaluate in future studies.

In conclusion, this observational study shows some novel insights into the gastrointestinal physiology of IBS. MRI has proven to be a useful tool to assess small and large bowel as it is patient acceptable and easily repeatable. Our findings will hopefully stimulate many new questions, in particular what effect commonly used drugs like antispasmodics and laxatives have in the gastrointestinal tract of IBS patients. These are all questions that MRI is well placed to answer. These future studies should also examine the response to treatments to determine how well colonic volumes explain the associated change in symptoms such as bloating and abdominal discomfort.

## Study Highlights


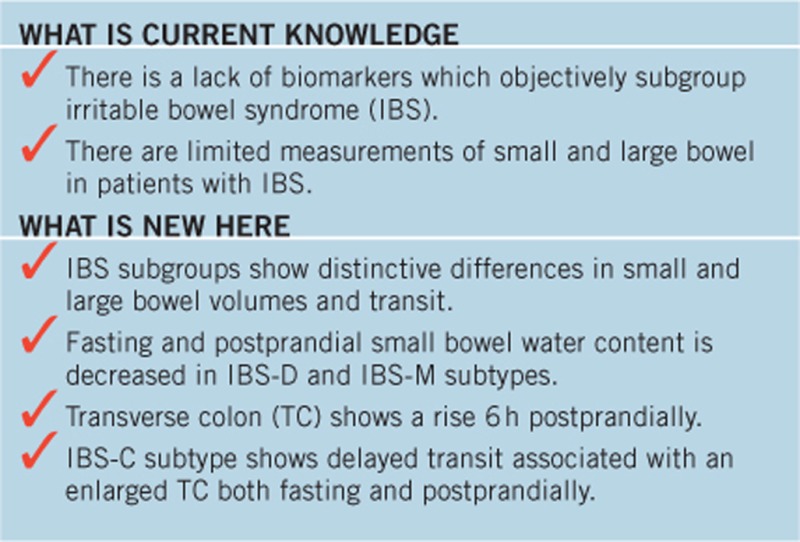


## Figures and Tables

**Table 1:** Demographics of HV and patients with IBS subtypes

**Median (IQR)**	**HV (*****n***** =34)**	**IBS-C (*****n***** =16)**	**IBS-D (*****n***** =30)**	**IBS-M (*****n***** =11)**	***P*** **value**
Age	24 (21–29)	36 (25–46)	44 (33–65)	34 (22–57)	
Female: male	15:19	15:1	18:12	7:4	χ^2^ 11.26, *P*=0.01
Anxiety score (0–21)	2.0 (1.0–4.0)	10.0 (6.3–12.8)^a^	6.5 (3.8–9.0)^a^	7.0 (4.0–12.0)^a^	<0.01 (Kruskal–Wallis)
Depression score (0–21)	3.5 (1.0–5.0)	4.5 (2.3–7.8)	3.0 (1.0–5.3)	5 (2.0–8.0)	0.18 (Kruskal–Wallis)
PHQSS12	2.0 (0–3.0)	8.5 (6.0–10.0)^a^	5.0 (2.8–8.0)^a^	7.0 (4.0–11.0)^a^	<0.01 (Kruskal-Wallis)
VSI	1.0 (0–7.5)	53.0 (40.5–64.0)^a^	40 (25.5–51.0)^a^	44.0 (32.0–51.0)^a^	<0.01 (Kruskal-Wallis)
IBSSS	29.5 (10–39)	340 (258–406)^a^	282 (191.5–363.0)^a^	265 (189–315)^a^	<0.01 (Kruskal–Wallis)
Perceived stress score	18.5 (8.3–29.5)	28.5 (24.3–30.0)	27.0 (15.5–31.5)	26.0 (16.0–33.0)	0.38 (Kruskal–Wallis)
Baseline average abdominal pain (0–10)	0 (0–0.1)	3.4 (2.5–3.9)^a^	3.3 (1.2–3.9)^a^	3.7 (2.9–7.6)^a^	<0.01 (Kruskal–Wallis)
Baseline average urgency (0–10)	0.1 (0–1.0)	1.9 (0.1–2.6)	4.7 (2.3–6.7)a,^b^	3.4 (1.4–4.7)^a^	<0.01 (Kruskal–Wallis)
Baseline average bloating (0–10)	0 (0–0.8)	5.2 (3.7–7.1)^a^	2.0 (0.6–5.8)^a^	4.4 (2.0–6.9)^a^	<0.01 (Kruskal–Wallis)
Baseline average daily stool frequency	1.1 (0.9–1.4)	1.0 (0.6–1.2)	2.5 (1.8–3.4)a,^b^	1.7 (1.3–2.9)^b^	<0.01 (Kruskal–Wallis)
Baseline average stool consistency (1–7)	3.8 (3.3–4.1)	2.1 (1.6–3.1)	5.4 (4.8–5.7)a,^b^	4.0 (3.6–4.4)b,^c^	<0.01 (Kruskal–Wallis)

HV, healthy volunteers; IBS, irritable bowel syndrome; IBS-C, IBS with constipation; IBS-D, IBS patients with diarrhea; IBS-M, IBS with mixed bowel habit; IQR, interquartile range; VSI, visceral sensitivity index.

a *P*<0.01 vs. HV.

b *P*≤0.03 vs. IBS-C.

c *P*<0.01 vs. IBS-D.

**Table 2:** Fasting and postprandial AUC (0–360 min) SBWC

	**HV**	**IBS-C**	**IBS-nonC**	***P*** **value**
Fasting SBWC (ml) at *t* =−45 min Median (IQR)	44 (15–70)	61 (23–87)	21 (10–42)a,^b^	<0.01 (Kruskal–Wallis)
AUC SBWC (l min) Mean (s.d.)^c^	23 (10)	19 (12)	14 (7)^a^	<0.01 (ANOVA)

ANOVA, analysis of variance; AUC, area under the curve; HV, healthy volunteers; IBS-C, IBS with constipation; IQR, interquartile range; SBWC, small bowel water content.

a *P*≤0.03 vs. HV.

b *P*≤0.03 vs. IBS-C.

c AUC for postprandial volume min ml (*t*=0 to *t*=360 min) ml min.

**Table 3:** Fasting and postprandial AUC (0–360 min) regional colonic volumes and colonic gas volumes

**Median (IQR)**	**HV**	**IBS-C**	**IBS-nonC**	***P*** **value**
Fasting AC (ml) at *t* =−45 min	194 (150–234)	217(191–268)	209 (147–248)	0.30 (Kruskal–Wallis)
Fasting TC (ml) at *t* =−45 min	165 (117–255)	253 (200–329)^a^	198 (106–270)^b^	<0.01 (Kruskal–Wallis)
Fasting DC (ml) at *t* =−45 min mean (s.d.)	143 (61)	153 (47)	114 (52)	0.02 (Kruskal–Wallis)
Fasting total colonic volume (ml) at *t* =−45 min mean (s.d.)	513 (174)	644 (148)^a^	498 (175)^b^	0.01 (ANOVA)
AUC AC l min^c^	67.6 (54.4–86.4)	76.8 (61.6–92.5)	65.7 (52.2–78.4)	0.21 (Kruskal-Wallis)
AUC TC volume l min mean (s.d.)^c^	67.9 (27.7)	96.9 (24.6)^a^	72.1 (25.8)^b^	< 0.01 (ANOVA)
AUC DC l min mean (s.d.)^c^	50.4 (21.2)	51.8 (18.1)	41.9 (17.0)	0.09 (ANOVA)
AUC total colonic volume l min^c^	179.7 (137.3–231.4)	224.0 (190.1–251.1)	172.0 (140.6–227.9)^b^	0.03 (Kruskal-Wallis)
Fasting total colonic gas (ml) at *t* =−45 min	16 (5–68)	17 (8–36)	13 (7–24)	0.69 (Kruskal-Wallis)
AUC total colonic gas l min^c^	9.8 (3.7–23.0)	8.8 (4.4–16.3)	7.71 (4.0–13.5)	0.70 (Kruskal-Wallis)

AC, ascending colon; AUC, area under the curve; HV, healthy volunteers; IBS-C, IBS with constipation; IQR, interquartile range; SBWC, small bowel water content; TC, transverse colon.

a *P*≤0.03 vs. HV.

b *P*≤0.03 vs. IBS-C.

c AUC for postprandial volume min ml (*t*=0 to *t*=360 min) ml min.

**Table 4:** OCTT and WGTT for HV and IBS subtypes

	**HV**	**IBS-C**	**IBS-D**	***P*** **value**
OCTT (min)	188 (135–262)	203 (154–266)	165 (116–244)	0.29 (Kruskal–Wallis)
WGTT (h)	34 (4–63)	69 (51–111)^a^	34 (17–78)	0.03 (Kruskal–Wallis)

HV, healthy volunteers; IBS, irritable bowel syndrome; IBS-C, IBS with constipation; IBS-D, IBS patients with diarrhea; OCTT, orocecal transit time; WGTT, whole-gut transit time.

a *P*≤0.03 vs. HV.

**Table 5:** Clinical correlations between anxiety, WGTT and orocecal transit for HV and IBS subtypes

**Correlation**	**HV**	**IBS-C**	**IBS-nonC**
Anxiety and OCTT	Spearman *r* =0.12	Pearson *r* =−0.39	Pearson *r* =−0.39
	*P* =0.51	*P* =0.14	*P* =0.02
Anxiety and WGTT	Spearman *r* =0.16	Pearson *r* =−0.69	Spearman *r* =0.21
	*P* =0.37	*P* ≤0.05	*P* =0.21

HV, healthy volunteers; IBS, irritable bowel syndrome; IBS-C, IBS with constipation; IBS-D, IBS patients with diarrhea; OCTT, orocecal transit time; WGTT, whole-gut transit time.
