# Supplementary material for: Distinct Abnormalities of Small Bowel and Regional Colonic Volumes in Subtypes of Irritable Bowel Syndrome Revealed by MRI
Source: Am J Gastroenterol. 2016 Dec 13;112(2):346–55. doi: 10.1038/ajg.2016.538 (PMC5318666; doi:10.1038/ajg.2016.538)
Supplement: Supplementary Figure 1 Legend [file ajg2016538x2.doc]

Supplementary Figure 1: Selected individual images fasting and at t=405 min illustrating the larger tansverse colon volume in IBS-C compared to other IBS subtypes.
